# A case of deep neck abscess treated with a disposable VSD wound care device: Case report and review of literature

**DOI:** 10.1097/MD.0000000000037397

**Published:** 2024-03-22

**Authors:** Ziyi Lu, Xinxin Zhang, Yixuan Huo, Shoukai Zhang

**Affiliations:** a The First Clinical Medicine College, Gansu University of Chinese Medicine, Lanzhou, Gansu, China; b Ningxia Medical University, Ningxia, China; c Department of Otolaryngology-Head and Neck Surgery, Gansu Provincial Hospital, Lanzhou, Gansu, China.

**Keywords:** deep neck abscess, treatment, vacuum sealing drainage

## Abstract

**Rationale:**

Vacuum sealing drainage is a novel technique for wound treatment that is characterized by adequate drainage and promotes wound healing. We report a case in which negative pressure sealing drainage was applied to treat a deep cervical abscess and achieved a good therapeutic effect.

**Patient concerns:**

The abscess in the neck will go down.

**Diagnoses:**

Deep neck abscess.

**Interventions:**

The usual surgical approach to treating this condition is to make a small incision to incise and drain the patient infected area where it is most visibly swollen or fluctuating, and to place a negative pressure drainage device.

**Outcomes:**

Eleven days after the operation, the patient neck recovered well, there was no infection in the operation area, and the patient was discharged from the hospital with improved symptoms.

**Lessons:**

This proves that the negative pressure closed drainage technique has potential in the treatment of deep neck abscesses and is also an effective choice in promoting wound healing, which is expected to bring better therapeutic effects to patients treated for deep neck abscesses.

## 1. Introduction

Deep cervical interstitial infections are a group of infectious septic diseases involving the deep cervical interstitium and cervical fascia.^[1]^ Severe cervical interstitial infections may lead to complications such as asphyxia, shock, and sepsis, and may even be life-threatening if proper management is not taken in time. The usual surgical approach to treating this condition is to make a small incision to incise and drain the patient infected area where it is most visibly swollen or fluctuating, and to place a negative pressure drainage device. Vacuum sealing drainage (VSD) is a novel technique for wound treatment that is characterized by adequate drainage and promotes wound healing. We report a case in which negative pressure sealing drainage was applied to treat a deep cervical abscess and achieved a good therapeutic effect.

## 2. Case report

**Figure 1.**
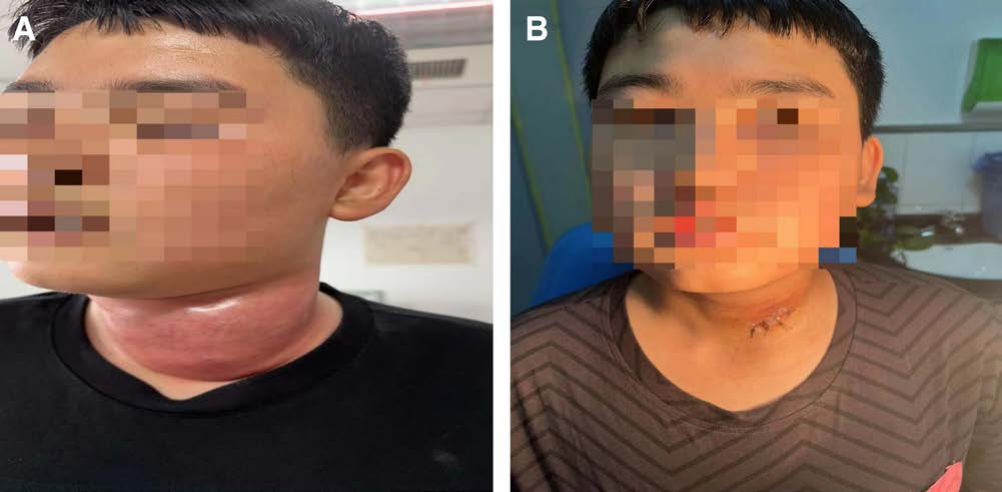
(A) Preoperative, (B) post-operative.

**Figure 2.**
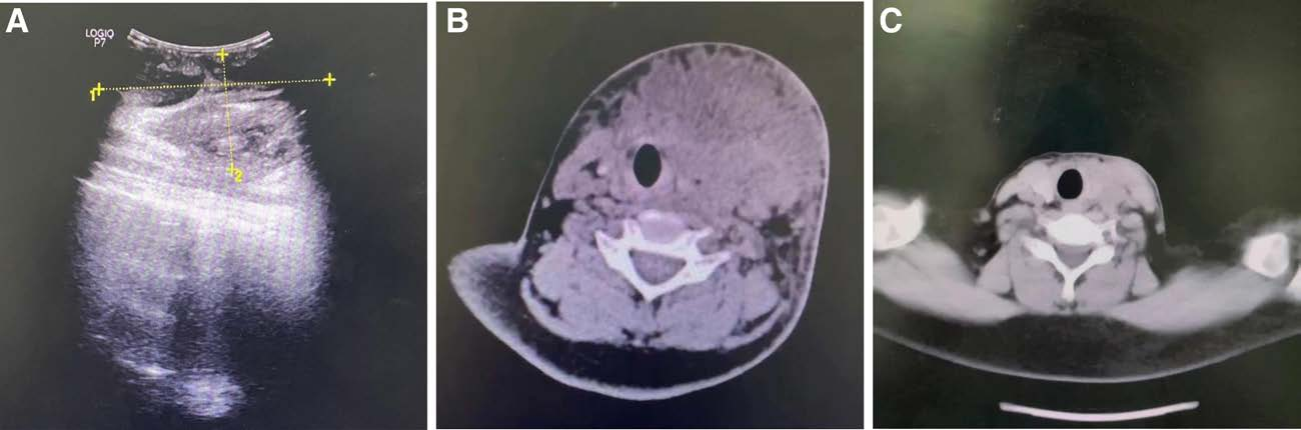
(A) Preoperative ultrasound, (B) preoperative CT, (C) postoperative CT.

**Figure 3.**
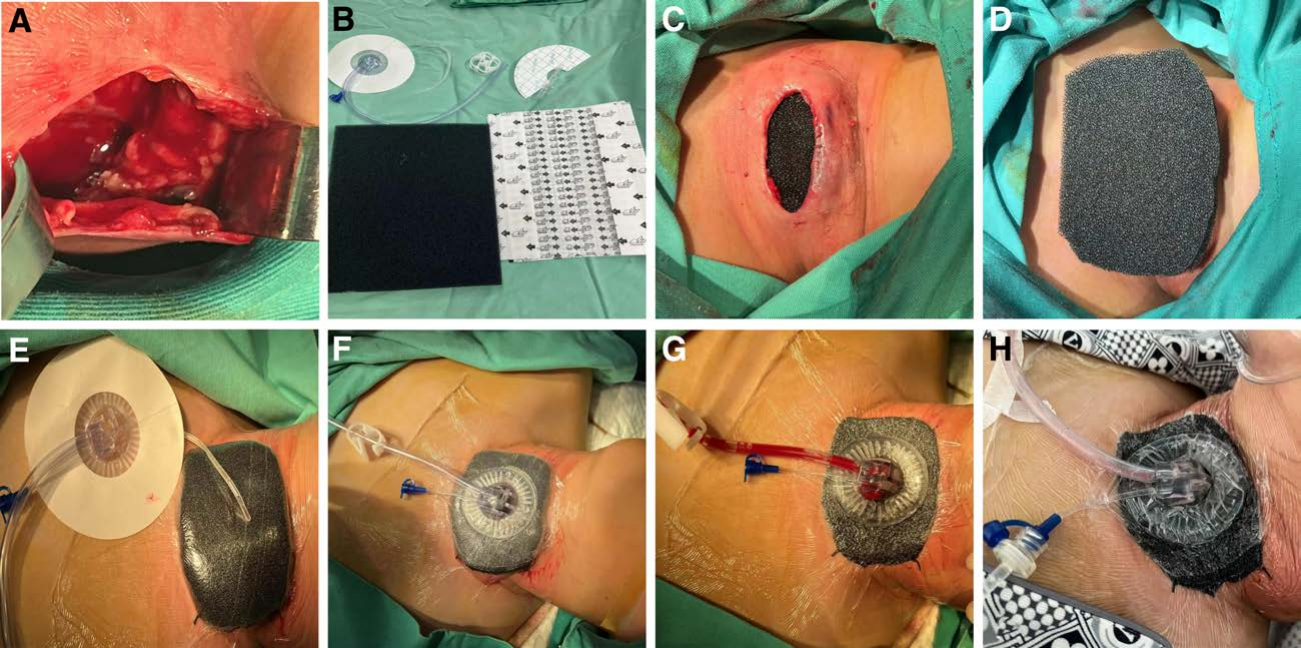
(A) Thorough cleaning of pus from the operative cavity, (B) VSD device, (C) cutting PVA foam to fill the entire wound, (D) cutting the PVA foam and covering the entire wound surface with it, (E) sealed with transparent film, (F) injection of sterile saline, (G) drainage patency, (H) 1 wk after surgery. PVA = polyvinyl-alcohol, VSD = vacuum sealing drainage.

The patient, male, 13 years old, was admitted to the hospital on 2023-06-22 with “neck swelling and fever for 10 days.” The patient presented with neck swelling (Fig. 1A) 10 days ago without any obvious cause, accompanied by congestion and redness, with neck pain, fever, coughing and sputum, nausea and vomiting, without headache and dizziness. There was no dysphagia. The patient was admitted to an outside hospital with an unknown diagnosis, and was treated with fluids and symptomatic treatment (specific treatment unknown), The above symptoms improved, but 2 days after the improvement, neck swelling and pain worsened again, accompanied by dysphagia. He was admitted to the hospital for further treatment. The patient was in good health and had no history of chronic diseases such as hypertension and diabetes mellitus. Examination: mouth opening was not restricted, pharynx was mildly congested, tonsils were large in the first degree without congestion, epiglottis, vocal folds, and pyriform fossa were generally normal, and the anterior left side of the neck was seen to be locally elevated, congested and swollen with a size of about 7 × 7 cm, accompanied by fluctuating sensation and obvious compression pain, and auxiliary examination: (superficial ultrasonography) subcutaneous neck was seen to be a liquid dark area of 101 × 51 mm, with unclear boundaries, and dense punctate echoes could be seen floating in the area, and the following were considered Subcutaneous abscess formation (Fig. 2A). Blood count + C-reactive protein: leukocyte counts 18.3 × 10^9^/L, lymphocyte percentage 13.0%, neutrophil count 14.4 × 10^9^/L, whole blood ultrasensitive C-reactive protein > 10.0 mg/L. Admission diagnosis: neck abscess? After admission, he was given fluticasone propionate nebulized inhalation with suspension 2g nebulized inhalation 2 times/d, cefmetazole sodium for injection 2g IV 2 times/d anti-infection treatment. Preoperative CT examination of the neck showed a soft tissue mass on the left side of the neck, which was considered an infectious lesion with multiple enlarged lymph nodes around the carotid sheaths bilaterally (Fig. 2B). After completing relevant preoperative examinations, neck exploration + incision and drainage of neck abscess was performed on 2023-06-22. During the operation, the skin was incised at the left side of the neck along the surface of the swelling with the dermatoglyphic pattern for about 2.5 cm, and a large amount of pus was seen to be overflowing (Fig. 3A), and some of the pus was taken and sent for bacterial culture and drug sensitivity test, and the suction device was used to suction out the pus to see an extensive pus cavity under the skin, and the pus cavity was repeatedly rinsed out with iodine-vapor diluted solution, and the necrotic tissues were cleaned up, and some of the tissues were seen to be missing under the skin, and there was a large amount of pus in the left side of the paratracheal space, which was dilated, cleaned up, and the paratracheal space was expanded and cleaned. pus, repeatedly flushing the operative cavity, adequate hemostasis in the operative area, and placing a VSD drainage device (Fig. 3B): according to the results of probing the incision, polyvinyl-alcohol foam was cut and covered the entire wound surface to ensure adequate contact with the wound surface(Fig. 3C and D); the outer layer of the bio-semi-permeable membrane was covered with the foam, and the bio-occlusive transparent membrane should be beyond the edge of the wound by 5 cm to achieve an airtightness(Fig. 3E); the VSD was sealed before use, and a connection was made to The VSD is sealed before use, connected to a flushing tube and suction tube, and the flushing tube is filled with sterile saline(Fig. 3F); the suction tube is connected to a negative pressure system located in the center of the tube wall, and the negative pressure is adjusted until the transparent layer on the outside of the foam of the VSD device is compressed and the foam is tightly bonded to the wound surface.^[2]^ The negative pressure was adjusted until the transparent layer on the outside of the VSD device foam was compressed and the foam was tightly bound to the wound surface, indicating that the negative pressure was sufficient, and continuous saline irrigation was used to generate negative pressure drainage(Fig. 3G). Postoperatively, piperacillin sodium 4.5g IV q8h and ornidazole 0.5g IV q12h were given as a combination of anti-infective treatment, and the patient was given routine dressing changes and diluted iodophor to flush the operative cavity, and the patient anterior neck pain was gradually relieved. The VSD drainage device was taken out 5d after surgery (Fig. 3H), and the surgical area was cleaned and sutured after good recovery. Postoperative 7d neck CT showed a soft tissue mass on the left side of the neck with peripheral edema, which was significantly reduced in scope compared with that of June 22; multiple enlarged lymph nodes around the bilateral carotid sheaths, some of which were slightly reduced compared with that of the previous one (Fig. 2C); 11 days after the operation, the patient neck recovered well, there was no infection in the operation area, and the patient was discharged from the hospital with improved symptoms (Fig. 1B).

## 3. Discussion

A deep cervical abscess is a disease in which the deep cervical fascia is invaded by a bacterial infection, and the local fascia and soft tissues become necrotic, lysed, and liquefied under the action of toxins or proteolytic enzymes released from the bacteria and inflammatory cells, and ultimately a deep cervical hiatus abscess is formed. The deep cervical space is surrounded by the deep cervical fascia and can be divided into the supraglottic, subglottic, and deep cervical spaces spanning the entire neck.^[3]^ The deep cervical space can be divided into suprahyoid, subhyoid, and across the whole neck. The fascia between adjacent deep cervical spaces is continuous, and lymphatic drainage occurs between the spaces. Once an infection occurs in one gap, it can spread to the adjacent gaps in a short time, such as odontogenic infection can involve the occlusal interspace outwardly, downwardly to the submandibular interspace, and backwardly to the parapharyngeal interspace; peri-tonsillar abscess can involve the parapharyngeal interspace; infection of the pre-tracheal interspace can involve the anterior superior mediastinum downwardly; and infection of the posterior pharyngeal interspace can involve the posterior mediastinum downwardly.^[4]^

## 4. Conclusion

Neck abscesses are treated based on surgical incision and drainage, which can adequately drain pus and remove necrotic tissue. In recent years VSD drainage technique has been a novel treatment for oral maxillofacial and neck infections.VSD converts open wounds into closed wounds, preventing contamination and infection.VSD is a punctate planar drainage, unlike previous drainage, the continuous high negative pressure drainage from VSD produces centripetal pulling force on the incision, which is superior to the centrifugal pressure produced by the traditional gauze tamponade. The effects of negative pressure wound therapy include rapid removal of interstitial fluid by negative pressure, reduction of local edema, and increased blood flow, which reduces the bacterial load on the tissues and decreases tissue edema.VSD improves the rate of microcirculatory blood flow in the wound, increases perfusion, accelerates necrotic resorption, increases the formation of granulation tissue, reduces the bacterial burden, promotes proinflammatory cytokines and proteases, and contracts hemostasis.^[5]^ Gu^[6]^ the results of the study by Xiang Gu and other researchers showed that the clinical effect of a modified VSD drainage device in treating neck abscess is good, which effectively promotes the healing of its wound, and reduces the number of dressing changes, reduces the pain of the patients, and achieves satisfactory results, which is worth to be clinically applied in the treatment of neck abscess; at the same time, it is necessary to reasonably use the antibiotic, actively treat the underlying diseases and concomitant diseases, keep the airway open, and prevent serious complications. This proves that the negative pressure closed drainage technique has potential in the treatment of deep neck abscesses and is also an effective choice in promoting wound healing, which is expected to bring better therapeutic effects to patients treated for deep neck abscesses. The limitation of this study is that there is only one case; in the future, multiple cases could be collected and compared to usual drains in a randomized way. Informed consent was secured from the legal guardian of kin for patients within this patient.

## Author contributions

**Conceptualization:** Shoukai Zhang.

**Funding acquisition:** Shoukai Zhang.

**Supervision:** Yixuan Huo.

**Writing – original draft:** Ziyi Lu.

**Writing – review & editing:** Xinxin Zhang.
